# Vaccine-induced antibodies linked to bovine neonatal pancytopenia (BNP) recognize cattle major histocompatibility complex class I (MHC I)

**DOI:** 10.1186/1297-9716-42-97

**Published:** 2011-08-30

**Authors:** Fabian Deutskens, Benjamin Lamp, Christiane M Riedel, Eveline Wentz, Günter Lochnit, Klaus Doll, Heinz-Jürgen Thiel, Till Rümenapf

**Affiliations:** 1 Institute of Virology, Faculty of Veterinary Medicine, Justus-Liebig-University Giessen, Germany; 2 Institute of Biochemistry, Justus-Liebig-University Giessen, Germany; 3 Clinic for Ruminants, Faculty of Veterinary Medicine, Justus-Liebig-University Giessen, Germany

## Abstract

A mysterious disease affecting calves, named bovine neonatal pancytopenia (BNP), emerged in 2007 in several European countries. Epidemiological studies revealed a connection between BNP and vaccination with an inactivated vaccine against bovine virus diarrhea (BVD). Alloantibodies reacting with blood leukocytes of calves were detected in serum and colostrum of dams, which have given birth to calves affected by BNP. To understand the linkage between vaccination and the development of alloantibodies, we determined the antigens reacting with these alloantibodies. Immunoprecipitation of surface proteins from bovine leukocytes and kidney cells using sera from dams with a confirmed case of BNP in their gestation history reacted with two dominant protein species of 44 and 12 kDa. These proteins were not detected by sera from dams, free of BVDV and not vaccinated against BVD, and from sera of animals vaccinated with a different inactivated BVD vaccine. The 44 kDa protein was identified by mass spectrometry analysis as MHC I, the other as β-2-microglobulin. The presence of major histocompatibility complex class I (MHC I) in the vaccine was confirmed by Western blot using a MHC I specific monoclonal antibody. A model of BNP pathogenesis is proposed.

## Introduction

A mysterious hemorrhagic disease of cattle emerged in 2007 affecting solely newborn calves [1]. First named "blood sweating", "hemorrhagic diathesis" and "bleeding calf syndrome" it was finally designated "bovine neonatal pancytopenia" (BNP) at the Satellite Symposium of the European Buiatric Congress in 2009 [2]. During the last years an increasing number of calves were affected by this syndrome. BNP cases were reported for several breeds and both genders are affected equally. Reports of BNP affected calves are known from several European countries including France, Germany, United Kingdom, Ireland, Netherlands, Belgium, Luxembourg, Italy, and Spain [2,3]. The disease is unknown in countries which do not vaccinate against bovine virus diarrhea virus (BVDV) like Denmark, Austria, and Switzerland [4].

BNP is characterized by severe external and internal hemorrhages. Clinical studies have shown that the bleedings are caused by a massive thrombocytopenia, usually connected with a severe leukopenia and depletion of bone marrow cells, the latter may result in complete aplasia [1,5-7]. Mortality is up to 90% in affected calves; mild to subclinical manifestations are rarely observed [8].

In the past, bleeding disorders in cattle due to thrombocytopenia have been described primarily as a consequence of intoxications and viral infections, but sporadic cases were also linked to bacterial septicemia, hereditary diseases or immune mediated processes [9]. Several hypotheses have been put forward with regard to the etiology of BNP, such as bacterial and virus infections (BVDV, bluetongue virus, porcine circovirus 2 [9]) or intoxications but could not be confirmed [1,8,10]. A genetic etiology of BNP was discussed, but BNP appearance showed no link to certain genotypes. Epidemiological studies showed that mutations in coagulation factor XI [11] or certain MHC class II haplotypes [12] were not associated with BNP outbreaks. In BNP affected calves, the often dramatic decline of thrombocyte and leukocyte counts within the first hours after ingestion of colostrum, together with the characteristic bone marrow findings (panmyelophthisis, phagocytic bone marrow macrophages) led to the assumption of an immune mediated process [13,14].

The occurrence of BNP is correlated with vaccination with an inactivated bovine virus diarrhea virus (BVDV) vaccine termed PregSure^® ^BVD (Pfizer, Karlsruhe, Germany) [4]. The vaccine contains cytopathogenic BVDV type 1 (strain 5960) grown on a bovine kidney cell line and as adjuvant Quil A, cholesterol, amphigen base and liquid paraffin (Procision-A™) [15]. Quil A is a mixture of saponins, which are extracted from the bark of the tree *Quillaja saponaria *Molina. Quil A forms immune-stimulatory complexes (ISCOMS) together with the other components of the adjuvant and antigens [16,17]. ISCOMS efficiently induce both, antigen-specific antibodies (IgG1 and IgG2) as well as T-cell response (Th1 and Th2) [18,19]. PregSure^® ^BVD was introduced in 2005. After growing evidence for a connection between the use of the vaccine and the occurrence of BNP the vaccine was retracted from the market by the manufacturer in 2010. It was speculated that vaccination with PregSure^® ^BVD induces alloantibodies which are transmitted via colostrum to the calves. According to this model antigens of the vaccine elicit antibodies that bind to peripheral blood cells as well as to the stem cells in the bone marrow of neonates, which elevated cytophagocytosis of opsonised cells by bovine macrophages [14,20].

Friedrich et al. demonstrated that BNP can be reproduced in healthy calves by transmission of colostrum from dams, which have given birth to at least one calf with confirmed BNP [4]. Alloantibodies (also called isoantibodies) were found in colostrum and serum of BNP-dams with exhibited reactivity with leukocytes from susceptible calves [20-22]. According to our definition BNP-dams are animals, which have a common vaccination history with PregSure^® ^BVD and have given birth to at least one calf with confirmed BNP syndrome. Non-BNP-dams are animals which were also vaccinated with PregSure^® ^BVD, but in their gestation history no case of BNP appeared. Susceptible calves are defined as newborns whose leukocytes bind alloantibodies from BNP-dam sera [21,22].

In Europe, over 4000 confirmed BNP cases were reported [20]. This reveals, in relation to 13 million sold doses of PregSure^® ^BVD in Europe [23] that the number of vaccinated dams with healthy offspring (non-BNP-dams) exceeds by far the number of dams whose offspring is affected by BNP. Apart from blood-group specific antigens an induction of alloantibodies has not yet been documented for a vaccine, so far [24-26].

Elucidation of BNP pathogenesis requires the identification of antigen(s) targeted by the alloantibodies. Here we report on the identification of MHC I as antigen specifically reacting with sera from BNP-dams.

## Materials and methods

### MDBK cells and primary bovine leukocytes

Madin-Darby bovine kidney cells (ATCC-CCL22) were grown in Dulbecco's modified Eagle's medium (DMEM) supplemented with 10% heat-inactivated fetal calf serum (FCS). The cells were maintained at 37°C and 5% CO_2 _for propagation.

Isolation of leukocytes was performed as described before [21]. Briefly, 50 mL of freshly collected blood samples were centrifuged (5 min with 2000 × *g*), the buffy coat was collected and washed with PBS. Contaminating erythrocytes were lysed by an incubation for 5 min in 5 mL lysis buffer (155 mM NH_4_Cl, 10 mM KHCO_3_, 0.13 mM EDTA) and the remaining leukocytes were harvested by centrifugation (5 min with 2000 × *g*). For quality control, the obtained leukocytes were counted and membrane integrity was assessed using trypan blue staining.

### Animals and serum samples

For serum preparation, peripheral blood samples were obtained from bovines at the Clinic for Ruminants, Justus-Liebig-University, Giessen. Different groups of blood donors were selected: BNP-dam sera (Group 1, *n *= 12) originated from dams, which had at least received a primary vaccination (two injections) with PregSure^® ^BVD and had given birth to at least one confirmed BNP-calf. For this study we considered "confirmed BNP-calves" as animals that (i) received colostrum only from their own dam, (ii) developed clinical signs of BNP, and (iii) showed severe thrombocytopenia and leukopenia, together with (iiii) depletion of the bone marrow.

A second group of serum samples originated from animals vaccinated with a different inactivated BVDV vaccine (Bovilis^® ^BVD, Intervet, Boxmeer, The Netherlands) (Group 2, *n *= 8), which were kindly provided by the Institute for Hygiene and Infectious Diseases of Animals, Justus-Liebig-University, Giessen. A third group of serum donors came from two farms with a BVDV free status. These dams were not vaccinated against BVD (Group 3, *n *= 8) and had no neutralizing antibodies against BVDV.

Additional blood samples were collected from PregSure^® ^BVD vaccinated animals from two farms, that had no reported case of BNP in their gestation history (non-BNP-dams) (Group 4, *n *= 4).

Sodium citrate was added in a proportion of 1:10 to the blood samples to prevent coagulation during retrieval. For serum production the anticoagulation effect of citrate was reversed by the addition of calcium chloride in a proportion of 1:300. Coagulation was allowed for 12 h at room temperature and blood samples were centrifuged (20 min at 2500 × *g*) to obtain the serum.

### Monoclonal antibodies and reagents

For the detection of cattle MHC class I the mouse monoclonal antibody IL-A88 was employed [27]. Streptavidin-HRP-conjugate, HRP-coupled goat anti-mouse antibodies were purchased from Dianova (Hamburg, Germany).

### Protein isolation from the PregSure^® ^BVD vaccine

The total protein content of 1 mL PregSure^® ^BVD vaccine was prepared by acetone precipitation. Briefly, 1 mL of the vaccine and 4 mL of ice cold acetone were mixed and incubated for two hours on ice. After centrifugation, the protein pellet was washed with n-hexane and ether to gain the vaccines proteome extricated from lipid contamination.

### Labelling of cell surface proteins and immunoprecipitation

Cell surface proteins of 2 × 10^6 ^MDBK cells or 2 × 10^6 ^bovine leukocytes were labelled with thiol cleavable EZ-Link Sulfo-NHS-LC-Biotin (Thermo Fisher Scientific, Rockford, IL, USA) according to manufacturer's recommendation. Briefly, the cells were washed with phosphate buffered saline (PBS) and labelling was performed for 30 min at 4°C. The labelling medium was then removed, biotinylation was stopped by incubation with 100 mM glycine and cells were washed and resuspended in 1 mL PBS. 300 μL of the respective undiluted bovine serum were added to each cell preparation and incubated for 90 min at 4°C on an agitator. Afterwards the cells were washed with PBS to eliminate unbound serum antibodies. Cells were lysed in 1 mL Triton X-100 buffer (1% Triton X-100, 150 mM NaCl, 50 mM Tris-HCl, 5 mM EDTA) in an ultrasonic bath at 4°C for 10 min. Insoluble matter was removed by centrifugation at 10 000 × *g *for 10 min at 4°C. Bovine antibodies together with the bound cellular proteins were isolated from these lysate with 35 μL protein-G-sepharose bead slurry (GE Healthcare, Munich, Germany). After an incubation of 90 min at 4°C on an agitator, the sepharose was washed four times with 1.5 mL ice cold lysis buffer. Proteins were harvested in 25 μL of loading buffer (6 M urea, 62.5 mM Tris-HCl, 2% SDS, 10% glycerol, 0.025% bromophenol blue, 0.025% phenol red) and resolved by SDS-PAGE. To prevent cleavage of the biotin linker all gels were run under non-reducing conditions and visualized by Western blotting and streptavidin-peroxidase.

### SDS-PAGE and Western blotting analysis

Proteins were separated in 7.5% polyacrylamide-tricin gel systems [28]. After SDS-PAGE, proteins were transferred to a nitrocellulose membrane (Pall Corporation, Dreieich, Germany). The membrane was blocked with 10% (w/v) Roti Block (Carl Roth, Karlsruhe, Germany) in PBS with 0.1% Tween 20 (v/v). After incubation with primary antibodies, HRP-conjugated goat anti-mouse IgG was added. For biotinylated proteins, streptavidin-peroxidase concentrate was used in a dilution of 1:30.000. A chemiluminescence reagent (Western Lightning Plus, Perkin Elmer, Rodgau, Germany) and X-ray films were utilized for signal detection.

### PNGase F digestion for detection of protein glycosylation

BNP-dam sera immunoprecipitated MDBK lysate (5 μL) was mixed with 5 μL of G7 reaction buffer and NP40 from a PNGase F kit (New England Biolabs, Ipswich, MA, USA). PNGase F (5 μL) was added and incubation carried out for 2 h at 37°C. 7.5 μL of loading buffer were added, the preparation was heated up for 5 min at 95°C, resolved by SDS-PAGE and visualized by Western blotting and streptavidin-peroxidase.

### Identification of target antigens of BNP-dam sera alloantibodies by peptide mass fingerprinting

For quantitative preparation of target antigens an upscaled immunoprecipitation was performed essentially as described above. For this purpose, 3 × 10^7 ^biotinylated MDBK cells were incubated with 3 mL of serum (in a total of 10 mL PBS) for 90 min at 4°C. Unbound antibodies were removed by repeated washes with PBS and cells were lysed in Triton X-100 buffer. Bovine IgG along with the target antigens was purified with 100 μL protein-G-sepharose bead slurry. Proteins were separated by SDS-PAGE and stained using coomassie brilliant blue G-250 (1% coomassie G-250 (w/v), 40% methanol (v/v), and 10% acetic acid (v/v)). The bands of interest (44 kDa and 12 kDa) were excised. After reduction with dithiothreitol (DTT) and carbamidomethylation, proteins were digested with trypsin. Matrix-assisted laser desorption ionization time-of-flight mass spectrometry (MALDI-TOF-MS) was performed on an Ultraflex I TOF/TOF mass spectrometer (Bruker Daltonics, Bremen, Germany) equipped with a nitrogen laser and a LIFT-MS/MS facility. The instrument was operated in the positive-ion reflectron mode using 2,5-dihydroxybenzoic acid and methylendiphosphonic acid (both from Sigma-Aldrich, St.Louis, MO, USA) as matrix. Sum spectra consisting of 200-400 single spectra were acquired. For data processing and instrument control the Compass 1.1 software package consisting of FlexControl 2.4, FlexAnalysis 3.0 and BioTools 3.0 was used.

Proteins were identified by MASCOT peptide mass fingerprint search using the NCBInr database. The search was restricted to bovine proteins, a mass tolerance of 75 ppm was allowed and carbamidomethylation of cysteine as global modification and oxidation of methionine as variable modification were used.

## Results

### Precipitation of cell surface antigens from MDBK cells with BNP-dam sera

It has recently been described that BNP alloantibodies react with intact leukocytes of susceptible calves [20-22]. We therefore selectively labelled cell surface proteins with a membrane impermeable biotinylation reagent and incubated with sera of different dams. After incubation, washing and lysis of the cells antibodies bound to antigens on the cells were affinity purified using protein-G-sepharose. Antigen was eluted with a buffer containing 2% SDS and 6 M urea, subjected to SDS-PAGE and transferred to nitrocellulose. Signals of biotinylated proteins were revealed using HRP conjugated streptavidin.

**Figure 1:**
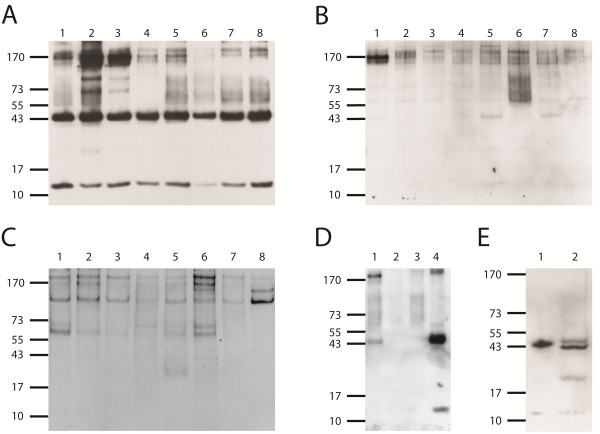
**Reactivity of sera from different groups of animals with biotinylated MDBK lysate in immunoprecipitation**. Figure 1A: Reactivity of sera from eight BNP-dams (Group 1), which were previously vaccinated with PregSure^® ^BVD, was tested with cell surface biotinylated MDBK lysate. For all experiments gel electrophoresis was employed under non-reducing conditions to prevent a cleavage of the biotin linker. Figure 1B: Eight sera of dams, which were vaccinated with a different inactivated BVD-vaccine (Bovilis^® ^BVD, Intervet) (Group 2) were tested for their reactivity with cell surface biotinylated MDBK lysate. Figure 1C: Eight sera of dams which were not vaccinated against BVD (Group 3), and originated from farms free of BVD, were tested for their reactivity with cell surface biotinylated MDBK lysate. Figure 1D: Four sera of dams vaccinated with PregSure^® ^BVD previously but without known BNP case in their offspring history (non-BNP-dams) (Group 4) were tested for alloantibodies recognizing protein species from cell surface biotinylated MDBK lysate (lane 1-4). Figure 1E: Deglycosylation of precipitate (from Figure 1A) with PNGase F. Lane 1: native precipitate. Lane 2: PNGase F digested precipitate.

It appeared likely that the induction of alloantibodies by the PregSure^® ^BVD vaccine is due to antigen(s) derived from bovine kidney cells that are described for propagation of the vaccine virus. As we could not obtain further details about this cell line we assumed that it is derived from Mabin-Darby bovine kidney (MDBK) cells (ATCC-CCL22) that are most commonly applied for the propagation of BVDV. Surface proteins of MDBK cells were labelled and used for immunoprecipitation. Sera from four different groups of animals were tested for their reactivity with biotinylated cell surface proteins of MDBK cells. Group 1 consisted of sera of twelve dams from two different farms, all of which had been vaccinated with PregSure^® ^BVD and had at least one confirmed case of BNP in gestation history (BNP-dam). All sera from this group precipitated antigens of approximately 44 kDa and 12 kDa (reactivity of eight sera shown in Figure 1A). For these two proteins only slight differences with regard to the signal strength were observed. The signal intensity of both proteins appeared to be connected, individuals with a strong signal for the 44 kDa protein showed also a strong signal intensity for the 12 kDa protein and vice versa. Group 2 consisted of eight dams from one farm without BNP history that had been vaccinated with a different BVD vaccine (Bovilis^® ^BVD, Intervet). These sera did not show apparent reactivity with cell surface proteins (Figure 1B) except for two sera that led to weak signal with proteins of apparent molecular weight of 44 and 12 kDa (Figure 1B, lanes 5+7). As additional negative control eight sera of dams that had neither been vaccinated against BVD, nor had been infected with BVDV (Group 3), were included in the experiment. No reactivity with the 44 kDa and 12 kDa proteins from biotinylated MDBK cells was observed with antibodies of these animals (Figure 1C).

As only a subset of PregSure^® ^BVD vaccinated dams give birth to BNP-calves, antibodies of non-BNP-dams vaccinated with PregSure^® ^BVD (Group 4) were analyzed with surface biotinylated MDBK cells using the above mentioned methods. From four dams repeatedly vaccinated with PregSure^® ^BVD, sera of two animals precipitated the 44 kDa and 12 kDa proteins observed previously (Figure 1D, lanes 1+4; see discussion), the other two showed no reactivity (Figure 1D, lanes 2+3).

To determine the glycosylation status of the recognized proteins PNGase F digestion was performed on BNP-dam serum precipitated MDBK cell surface proteins. For the 44 kDa protein a reduction of the apparent molecular mass of approximately 4 kDa was observed (Figure 1E, lane 2) compared to undigested protein (Figure 1E, lane 1) revealing N-glycosylation of this protein. No mass shift was detected for the 12 kDa protein.

### Antibodies of BNP dams recognize 44 kDa and 12 kDa cell surface antigens on primary leukocytes of susceptible calves and non-BNP-dams

**Figure 2:**
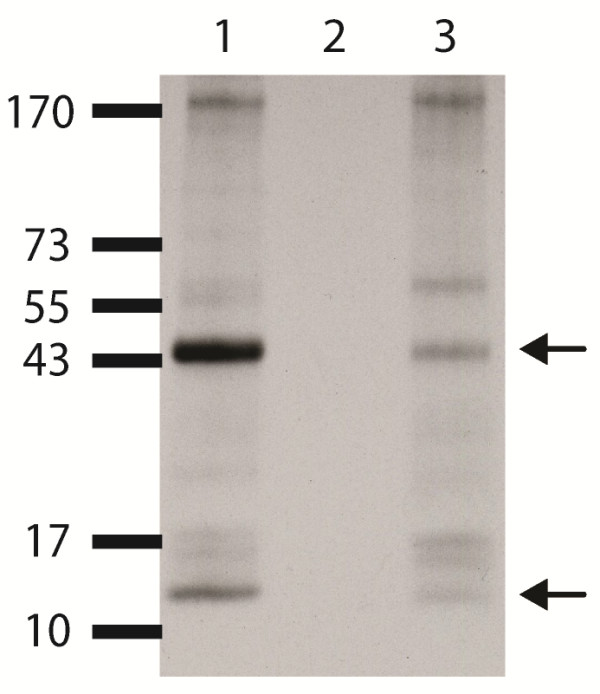
**Reactivity of serum from a BNP-dam with leukocytes from different animals**. Immunoprecipitation was performed with serum of a BNP-dam on cell surface biotinylated leukocytes from a susceptible calf (lane 1), a different BNP-dam (lane 2) and a non-BNP-dam, which was vaccinated with PregSure^® ^BVD, but was tested negative for MHC I alloantibodies (lane 3). The 44 kDa and 12 kDa protein (arrows) were only recognized in the susceptible calf and the non-BNP-dam.

Surface biotinylated leukocyte samples from BNP-dams (Figure 2, lane 2), from non-BNP-dams (vaccinated with PregSure^® ^BVD, lacking of antibodies against the 44 and 12 kDa protein) (Figure 2, lane 3), and from a BNP susceptible calf [21] (Figure 2, lane 1) were precipitated with serum from a BNP-dam. Antibodies of BNP-dam serum led to the detection of antigens apparently of the same molecular mass (44 kDa and 12 kDa) on leukocytes of the susceptible calf and non-BNP-dams, respectively. No reactivity or very weak reactivity of BNP-dam serum was observed with specific proteins from leukocytes from a different BNP-dam. The results were reproduced with two other sera from BNP-dams using the same leukocyte preparations (from susceptible calf, BNP-dams and non-BNP-dams) (data not shown).

### Identification of MHC I and β-2-microglobulin by peptide mass fingerprinting

**Figure 3:**
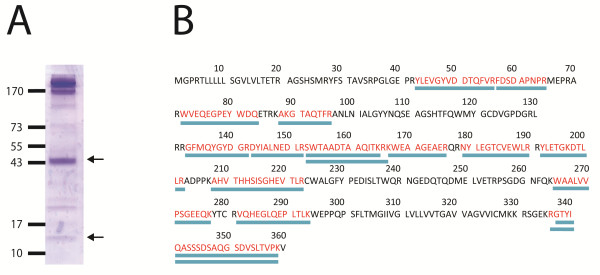
**MALDI-TOF peptide mass fingerprinting analysis**. Figure 3A: Coomassie brilliant blue G250 stained gel of BNP-dam serum precipitated MDBK lysate. Bands of interest (44 kDa and 12 kDa, arrows) were excised and analyzed by MALDI-TOF peptide mass fingerprinting. Figure 3B: MHC I [EMBL:AAZ73457.1 (http://www.ebi.ac.uk/ena/data/view/AAZ73457)] sequence coverage of detected peptides was 49.2% (blue bars). Two peptide masses (997.6 Da; 1127.6 Da) derived from the 12 kDa band, matched to tryptic peptides _103_HVTLEQPR and _68_SEQSDLSFSK of bovine β-2-microglobulin [EMBL:AAI18353.1 (http://www.ebi.ac.uk/ena/data/view/AAI18353)].

For the identification of the detected antigens, 3 × 10^7 ^biotinylated MBDK cells were incubated with a strongly reactive BNP-dam serum, and resulting immune complexes were purified with immobilized protein-G. The eluate was subjected to SDS-PAGE, and the gel was stained with coomassie brilliant blue G-250. The discrete protein bands occurring around 44 kDa and 12 kDa (Figure 3A) were excised, digested with trypsin and the released peptides subjected to MALDI-TOF mass spectrometry. 16 peptides of the 44 kDa protein matched with a sequence coverage of 49.2% to two cattle MHC class I variants [EMBL:AAZ73457.1 (http://www.ebi.ac.uk/ena/data/view/AAZ73457), EMBL:AAZ73463.1 (http://www.ebi.ac.uk/ena/data/view/AAZ73463)] (Figure 3B). According to the IPD database [29] these sequences correlate to BoLA-N*01101 (genomic DNA: [EMBL:DQ121184]) and BoLA-N*05001 (genomic DNA: [EMBL:DQ121190]), respectively. Two peptide masses (997.6 Da and 1127.6 Da) derived from the 12 kDa band, matched to tryptic peptides _103_HVTLEQPR and _68_SEQSDLSFSK, respectively, of bovine β-2-microglobulin [EMBL:AAI18353.1 (http://www.ebi.ac.uk/ena/data/view/AAI18353)] yielding a coverage of 15.3%.

### MHC I is a contaminant of the PregSure^® ^BVD vaccine

**Figure 4:**
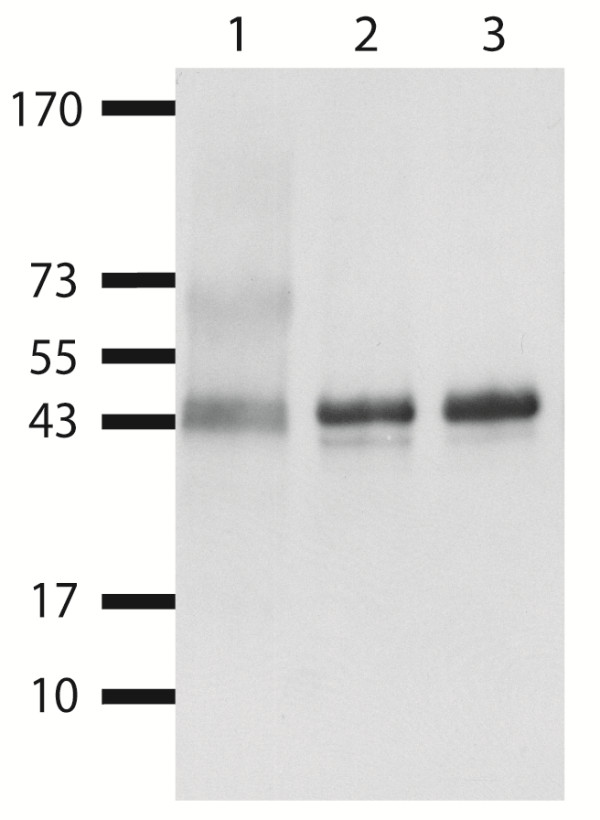
**Detection of MHC I in PregSure^® ^BVD vaccine**. Western blot analysis with the monoclonal antibody IL-A88 against bovine MHC I was applied to the PregSure^® ^BVD vaccine (lane 1). The antibody was further employed on native MDBK cell lysate (lane 2) and a precipitate from MDBK cells obtained with serum of a BNP-dam (lane 3).

If in fact the vaccine is responsible for the alloantibody induction against MHC I, the respective molecule has to be a constituent of the vaccine. To analyze its antigen composition the proteinaceous components of the vaccine were precipitated using acetone and subjected to immunoblot analyses. Using murine mAb against bovine MHC I (IL-A88), the 44 kDa protein was easily detectable in the PregSure^® ^BVD vaccine (Figure 4, lane 1). The same signal was observed with a lysate from MDBK cells (Figure 4 lane 2) that was employed as a control. Further to this IL-A88 was strongly reactive with the 44 kDa protein that was immunoprecipitated from MDBK cells using a serum from a BNP-dam (Figure 4, lane 3). This can be taken as independent evidence that the BNP sera and IL-A88 react with the same antigen, MHC I.

## Discussion

The key experiment for identification of bovine MHC I as an antigen detected by alloreactive antibodies was the labelling of cell surface proteins with biotin together with immunoaffinity purification using mild conditions. All sera from BNP-dams (*n *= 12) previously vaccinated with PregSure^® ^BVD reacted with apparently the same 44 kDa and 12 kDa antigens of MDBK cells (Figure 1A). In contrast sera from non vaccinated dams (*n *= 8, Figure 1c) or dams vaccinated with a different BVD vaccine (*n *= 8, Figure 1B) showed no or only a very weak reactivity. Additional cell surface antigens recognized by individual sera cannot be excluded from participation in BNP pathogenesis at this time.

For protein identification, alloantigens isolated by immunoprecipitation from MDBK cells were subjected to peptide mass fingerprinting analyses. The peptide mass fingerprints covered 49.2% of the bovine MHC I sequences (Figure 3B) of two variants of bovine MHC I, and 15.3% of the mature bovine β-2-microglobulin sequence (not shown). MHC I is a highly polymorphic, N-glycosylated, membrane bound protein that is transported to the cell surface in non-covalent association with β-2-microglobulin (MW: 12 kDa). MHC I molecules are present on all nucleated cells, but the expression levels are especially high on hematopoietic cells [30]. Functions of MHC I include the presentation of peptides (8-10 amino acids) from endogenous antigens to CD8+ T-cells at the cell surface. Heterologous MHC I molecules are involved in graft rejection [31]. According to the IPD database [29] there are at least 61 different alleles in cattle encoding MHC I. Six gene loci for MHC I are uniformly located on chromosome 23 resulting in a maximum of 12 different alleles in each individual animal [32]. In contrast, bovine β-2-microglobulin is invariable. Using monoclonal antibody IL-A88 directed against bovine MHC I a 44 kDa protein was detected in the PregSure^® ^BVD vaccine (Figure 4, lane 1). IL-A88 and BNP-dam sera both recognize the 44 kDa antigen from MBDK cells (Figure 4, lane 3).

**Figure 5:**
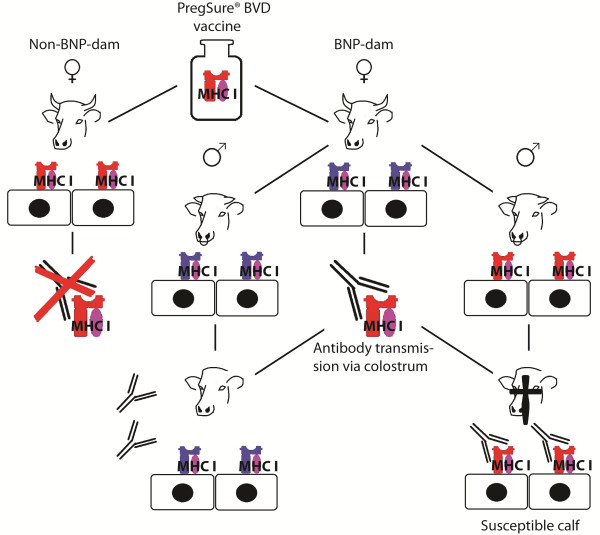
**Model of the BNP-etiology**. Dams vaccinated with PregSure^® ^BVD which carry the MHC I variant(s) of the vaccine on their cells (top left dam, MHC I colored in red) do not produce MHC I alloantibodies and their offspring does not develop BNP. If the MHC I variant(s) on the cells differ from MHC I variant(s) from the vaccine (top right dam, MHC I colored in blue) alloantibody production is induced. In this case the development of BNP depends on hereditary transmission of paternal MHC I variants. If the paternal MHC I variants are not recognized by the alloantibodies no BNP will occur (bottom left calf). BNP syndrome only appears if the paternal MHC I variants match with the MHC I variants present in the PregSure^® ^BVD vaccine (bottom right calf). Calves of both genders develop BNP equally as gene loci for MHC I are located on an autosome (chromosome 23).

The BNP syndrome has in most cases been reported for offspring of dams that received PregSure^® ^BVD vaccination. Interestingly BNP syndrome occurred only in a relatively small number of PregSure^® ^BVD vaccinated animals [20]. This indicates that factors in addition to the vaccine are relevant for the establishment of BNP. All tested BNP-dam sera recognized MHC I. From the four tested non-BNP-dams two sera did not show a reactivity with MHC I, although these dams were vaccinated with PregSure^® ^BVD (Figure 1D, lane 2 and 3). Whereas the two remaining sera of non-BNP-dams displayed clear reactivity with MHC I (Figure 1D, lane 1 and 4), but offspring of these dams was healthy in regard to BNP. Are these observations compatible with a role of antibodies to MHC I in BNP pathogenesis? PregSure^® ^BVD is produced by propagation of BVDV strain 5960 on bovine kidney cells and thus MHC I represents a contaminant. Whether the vaccine derived MHC I is antigenic in vaccinated dams must depend on the MHC I allotype constellation of the individual animal. If a vaccinated dam shares MHC I allotypes present in the PregSure^® ^BVD vaccine no immune response will ensue and consequently no BNP will occur. This is probably true for two PregSure^® ^BVD vaccinated animals that did not recognize MHC I from MDBK cells. Further evidence for this assumption is the presence of antigenetic MHC I variant(s) of BNP alloantibodies on non-BNP-dam leukocytes (Figure 2, lane 3). If a dam differs in its allotype repertoire it recognizes MHC I from the vaccine as foreign antigen and an immune response will be mounted. This is the case for all BNP-dams of this study as they all show reactivity with MHC I from MDBK cells (Figure 1A) and do not react with MHC I epitope(s) of leukocytes from other BNP-dams (Figure 2, lane 2). The occurrence of BNP is thus restricted to calves that share common MHC I epitope(s) with the PregSure^® ^BVD vaccine and originate from dams lacking this epitope(s). The MHC I allotype that renders calves susceptible (or unsusceptible) has to be of paternal origin. Calves carrying a paternally transmitted MHC I variant similar to that of the PregSure^® ^BVD vaccine will be targeted by maternal alloantibodies. Binding of the alloantibodies will lead to complement dependent lysis and/or cytophagocytosis [20], preferentially of cells expressing high levels of antigen. If the transmitted paternal allotype differs from MHC I in PregSure^® ^BVD no binding of the maternal alloantibodies to hematopoietic cells will occur and the calf will be unaffected. We therefore postulate that BNP occurrence is restricted to certain combinations of MHC I allotypes (Figure 5). It is currently not possible to predict whether the BNP susceptibility is conferred by a single or multiple MHC I alleles and whether these are rare or abundant in the population. Future studies will therefore aim at the determination of alloantigenic MHC I variants and their distribution in the cattle population. Cloning and expression of the respective MHC I genes will be required to define reactive MHC I variants. Using these approaches mapping of alloantibody specific epitopes on MHC I will be addressed in future experiments.

In cases of BNP only hematopoietic and bone marrow cells are clearly affected. However, MHC I is expressed on every nucleated cell. Why are other cells not destroyed? One possible explanation is the density of MHC I on the surface of cells, which is known to be high on hematopoietic cells [30]. Future investigations will determine amount and surface localization of MHC I in different cell populations particularly endothelial and epithelial cells.

According to our model alloantibodies against MHC I are responsible for the occurrence of BNP. It is generally assumed that BNP is a severe and in most cases lethal disease, with only a few calves surviving [1,5-7]. However recently a single herd with some BNP cases was thoroughly investigated with regard to blood parameters [33]. It turned out that a significant number of calves without clinical signs of BNP showed alterations in at least two of the three blood cell lineages. This observation leads to the assumption that the occurrence of BNP is probably dependent on the amount of alloantibodies against MHC I taken up with the colostrum. A low amount of such alloantibodies does probably not lead to clinical signs (as those are not known from the offspring of the two animals of Group 2, whose serum also showed a weak reaction with MHC I). Factors which could influence BNP occurrence are (i) the amount of colostrum taken up in the first 36 h of life, which differs from calf to calf [34], (ii) the individual titer of MHC I alloantibodies in a dam's colostrum arising from the number of boosts dams got with the PregSure^® ^BVD vaccine and the time point after vaccination. Occurrence presumably also depends on (iii) the affinity of alloantibodies to the MHC I variants of individual calves allotype.

Interestingly, a rare disease termed "neonatal alloimmune thrombocytopenia" (NAIT) occurring in human infants shows resemblance with clinical symptoms of BNP. The NAIT syndrome is caused by diaplacental transmission of maternal antibodies that are directed against paternal alloantigens on hematopoietic cells. While alloantibodies in most NAIT cases are directed against human platelet antigen (HPA) [35], some cases of NAIT have been linked to human leukocyte antigens (HLA), the human homologue to cattle MHC I (BoLA) [36]. In humans, antibodies can freely cross the placenta barrier so that alloantibodies can affect the fetus in utero. This is different in ruminants, because the placenta epitheliochorials is impermeable to immunoglobulins and maternal antibodies including BNP-alloantibodies are transmitted to the newborn exclusively in the first hours of life by uptake of colostral milk [21].

In the context of PregSure^® ^BVD the immune enhancing impact of the adjuvant Quil A has to be considered. Previous studies have shown, that PregSure^® ^BVD vaccination led to a robust immunity against BVD correlating with the induction of very high neutralizing antibody titers [37,38]. Therefore, it is conceivable that this special adjuvant induces a disproportionate immune response not only against viral antigens, but also against cellular alloantigens and may thereby be of pivotal importance in BNP induction.

The observation that the presence of MHC I in a vaccine probably causes a lethal disease in calves is relevant for inactivated vaccines in general. The use of a homologous production system (i.e. bovine cells for a bovine vaccine) bears potential risks for alloantibody induction and should therefore be avoided.

In conclusion, we have provided evidence that PregSure^® ^BVD vaccination leads to the induction of alloantibodies against MHC I which are probably linked to the pathogenesis of BNP. While it cannot be excluded that other alloantigens contribute to the development of BNP, MHC I is an excellent candidate. Future studies will include the reproduction of the disease by vaccination of purified components such as MHC I in combination with Quil A.

## Competing interests

The authors declare that they have no competing interests.

## Authors' contributions

FD, BL and TR carried out the experiments, participated in the data collection and analysis, and prepared the manuscript. CMR, EW, KD and HJT participated in the design of the study, draft of the manuscript and manuscript revision. GL participated in data collection (MALDI-MS) and data analysis. All authors read and approved the final manuscript.

## References

[B1] Friedrich A Rademacher G Weber B Kappe E Carlin A Assad A Sauter-Louis C Hafner-Marx A Büttner M Böttcher J Klee W Gehäuftes Auftreten von hämorrhagischer Diathese infolge Knochenmarkschädigung bei jungen Kälbern Tierärztl Umschau 2009 64 423 431 in German21889519

[B2] Buiatrie SFdB Klee W Abstracts Haemorrrhagic Diathesis in Calves Proceedings of the Satellite Symposium "Haemorrrhagic Diathesis in Calves" 2009 Société Francaise de Buiatrie 1 29 Marseille21861211

[B3] Armengol R Ponté D de Prado A Sierra M Casa M de las Heras M Uixera A García-Jalón J Síndrome dela diátesis hemorrágica del ternero (pancitopenia neonatal bovina) en Espana Boletín de anembe 2010 85 28 31 (in Spanish)21216839

[B4] Friedrich A Büttner M Rademacher G Klee W Weber BK Müller M Carlin A Assad A Hafner-Marx A Sauter-Louis CM Ingestion of colostrum from specific cows induces Bovine Neonatal Pancytopenia (BNP) in some calves BMC Vet Res 2011 7 10 10.1186/1746-6148-7-10 21333009 PMC3050708

[B5] Bell C Scott P Penny C Klee W Ten cases of "Bleeding Calf Syndrome" in a Scottish beef herd: Clinical signs, haematology and attempted treatment Proceedings of the Satellite Symposium "Haemorrrhagic Diathesis in Calves" 2009 Toulouse: Société Francaise de Buiatrie 17 Marseille21861211

[B6] Bell C Scott P Penny C Klee W Ten cases of "Bleeding Calf Syndrome" in a Scottish beef herd: post-mortem findings Proceedings of the Satellite Symposium "Haemorrrhagic Diathesis in Calves" 2009 Toulouse: Société Francaise de Buiatrie 21 Marseille21861211

[B7] Corbiere F Foucras G Lacroux C Meyer G Schelcher F Klee W Haemorrhagic diathesis syndrome: clinical and epidemiological findings of 48 suspected cases in France, 2007-2009 Proceedings of the Satellite Symposium "Haemorrrhagic Diathesis in Calves" 2009 Toulouse: Société Francaise de Buiatrie 11 13 Marseille21861211

[B8] Pardon B Steukers L Dierick J Ducatelle R Saey V Maes S Vercauteren G De Clercq K Callens J De Bleecker K Deprez P Haemorrhagic diathesis in neonatal calves: an emerging syndrome in Europe Transbound Emerg Dis 2010 57 135 146 10.1111/j.1865-1682.2010.01098.x 20202175

[B9] Kappe EC Halami MY Schade B Alex M Hoffmann D Gangl A Meyer K Dekant W Schwarz BA Johne R Buitkamp J Böttcher J Müller H Bone marrow depletion with haemorrhagic diathesis in calves in Germany: characterization of the disease and preliminary investigations on its aetiology Berl Munch Tierarztl Wochenschr 2010 123 31 41 20135908

[B10] Willoughby K Gilray J Maley M Dastjerdi A Steinbach F Banks M Scholes S Howie F Holliman A Baird P McKillen J Lack of evidence for circovirus involvement in bovine neonatal pancytopenia Vet Rec 2010 166 436 437 20364016 10.1136/vr.c1685

[B11] Krappmann K Weikard R Gerst S Wolf C Kuhn C A genetic predisposition for bovine neonatal pancytopenia is not due to mutations in coagulation factor XI Vet J in press 10.1016/j.tvjl.2010.10.007 21087874

[B12] Ballingall KT Nath M Holliman A Laming E Steele P Willoughby K Lack of evidence for an association between MHC diversity and the development of bovine neonatal pancytopenia in Holstein dairy cattle Vet Immunol Immunopathol 2011 141 128 132 10.1016/j.vetimm.2011.01.017 21353314

[B13] Doll K Wenzel L König M Krankheitsverlauf bei Kälbern mit hämorrhagischer Diathese Congr Proc 2nd Meeting of the "Deutsche Buiatrische Gesellschaft", Berlin 2010 20 23

[B14] Bauer N Wenzel L Moritz A Doll K Entwicklung der Blutbild- und Knochenmarksbefunde bei Kälbern mit hämorrhagischer Diathese Cong Proc 2nd meeting of the German Buiatrics Association; DVG-Service GmbH; Berlin 2008 23 29

[B15] Summary of Product Characteristics, PregSure BVD http://www.vmd.defra.gov.uk/ProductInformationdatabase/

[B16] Lendemans DG Myschik J Hook S Rades T Cationic cage-like complexes formed by DC-cholesterol, Quil-A, and phospholipid J Pharm Sci 2005 94 1794 1807 10.1002/jps.20394 15986471

[B17] Pham HL Ross BP McGeary RP Shaw PN Hewavitharana AK Davies NM Saponins from Quillaja saponaria Molina: isolation, characterization and ability to form immuno stimulatory complexes (ISCOMs) Curr Drug Deliv 2006 3 389 397 10.2174/156720106778559092 17076641

[B18] Sjolander A van't Land B Lovgren Bengtsson K Iscoms containing purified Quillaja saponins upregulate both Th1-like and Th2-like immune responses Cell Immunol 1997 177 69 76 10.1006/cimm.1997.1088 9140097

[B19] Platt R Coutu C Meinert T Roth JA Humoral and T cell-mediated immune responses to bivalent killed bovine viral diarrhea virus vaccine in beef cattle Vet Immunol Immunopathol 2008 122 8 15 10.1016/j.vetimm.2007.11.009 18190971

[B20] Bastian M Holsteg M Hanke-Robinson H Duchow K Cussler K Bovine Neonatal Pancytopenia: Is this alloimmune syndrome caused by vaccine-induced alloreactive antibodies? Vaccine 2011 29 5267 5275 10.1016/j.vaccine.2011.05.012 21605614 PMC7126856

[B21] Bridger PS Bauerfeind R Wenzel L Bauer N Menge C Thiel HJ Reinacher M Doll K Detection of colostrum-derived alloantibodies in calves with bovine neonatal pancytopenia Vet Immunol Immunopathol 2011 141 1 10 10.1016/j.vetimm.2011.01.001 21272941

[B22] Pardon B Stuyven E Stuyvaert S Hostens M Dewulf J Goddeeris BM Cox E Deprez P Sera from dams of calves with bovine neonatal pancytopenia contain alloimmune antibodies directed against calf leukocytes Vet Immunol Immunopathol 2011 141 293 300 10.1016/j.vetimm.2011.02.017 21440315

[B23] Pfizer, Pfizer animal health stops sale of cattle BVD vaccine in the EU 2010 http://www.pfizerah.co.uk/sites/sante-animale/UK-English/News/Pages/Pregsure_BVD_press_release.aspx

[B24] Luther DG Cox HU Nelson WO Screening for neonatal isohemolytic anemia in calves Am J Vet Res 1985 46 1078 1079 4003882

[B25] Osterhoff DR de Vos AJ Isoimmune blood group antibodies in cattle after the use of a blood vaccine J S Afr Vet Assoc 1977 48 137 139 410928

[B26] Stormont C Neonatal isoerythrolysis in domestic animals: a comparative review Adv Vet Sci Comp Med 1975 19 23 45 1108617

[B27] Toye PG MacHugh ND Bensaid AM Alberti S Teale AJ Morrison WI Transfection into mouse L cells of genes encoding two serologically and functionally distinct bovine class I MHC molecules from a MHC-homozygous animal: evidence for a second class I locus in cattle Immunology 1990 70 20 26 2354859 PMC1384076

[B28] Schagger H von Jagow G Tricine-sodium dodecyl sulfate-polyacrylamide gel electrophoresis for the separation of proteins in the range from 1 to 100 kDa Anal Biochem 1987 166 368 379 10.1016/0003-2697(87)90587-2 2449095

[B29] Robinson J Mistry K McWilliam H Lopez R Marsh SG IPD--the Immuno Polymorphism Database Nucleic Acids Res 2010 38 863 869 http://www.ebi.ac.uk/ipd/mhc/bola 10.1093/nar/gkp879 PMC2808958 19875415

[B30] Agrawal S Kishore MC MHC class I gene expression and regulation J Hematother Stem Cell Res 2000 9 795 812 10.1089/152581600750062237 11177592

[B31] Clatworthy MR Espeli M Torpey N Smith KGC The generation and maintenance of serum alloantibody Curr Opin Immunol 2010 22 669 681 10.1016/j.coi.2010.08.018 20932734 PMC4148597

[B32] Birch J Codner G Guzman E Ellis SA Genomic location and characterisation of nonclassical MHC class I genes in cattle Immunogenetics 2008 60 267 273 10.1007/s00251-008-0294-2 18431566

[B33] Witt K Weber CN Meyer J Buchheit-Renko S Müller KE Haematological analysis of calves with bovine neonatal pancytopenia Vet Rec in press 10.1136/vr.d4357 21795309

[B34] Vasseur E Rushen J de Passille AM Does a calf's motivation to ingest colostrum depend on time since birth, calf vigor, or provision of heat? J Dairy Sci 2009 92 3915 3921 10.3168/jds.2008-1823 19620674

[B35] Kaplan C Foetal and neonatal alloimmune thrombocytopaenia Orphanet J Rare Dis 2006 1 39 10.1186/1750-1172-1-39 17032445 PMC1624806

[B36] Gramatges MM Fani P Nadeau K Pereira S Jeng MR Neonatal alloimmune thrombocytopenia and neutropenia associated with maternal human leukocyte antigen antibodies Pediatr Blood Cancer 2009 53 97 99 10.1002/pbc.21979 19229975

[B37] Raue R Harmeyer SS Nanjiani IA Antibody responses to inactivated vaccines and natural infection in cattle using bovine viral diarrhoea virus ELISA kits: assessment of potential to differentiate infected and vaccinated animals Vet J 2011 187 330 334 10.1016/j.tvjl.2009.12.013 20074985

[B38] Salt S Antonis A Peters A Brune A Jahnecke S Traeder W PregSure® BVD - eine neue inaktivierte BVD-Vakzine. Breite Kreuzneutralisation von europäischen BVDV-Typ-1-und-Typ-2-Stämmen und signifikante Verbesserung der Fertilität nach Testinfektionen Tierarztl Prax Ausg G Grosstiere Nutztiere 2004 32 4 91 195 in German

